# Host genotype-specific microbiota do not influence the susceptibility of *D*. *magna* to a bacterial pathogen

**DOI:** 10.1038/s41598-018-27681-x

**Published:** 2018-06-20

**Authors:** Marilou P. Sison-Mangus, César M. J. A. Metzger, Dieter Ebert

**Affiliations:** 1 Department of Ocean Sciences, University of California Santa Cruz, 1156 High Street, Santa Cruz, CA 95064 USA; 2 Department of Environmental Sciences, Zoology, University of Basel, Vesalgasse 1, 4051 Basel, Switzerland; 3 Spiez Laboratory, Austrasse, 3700 Spiez, Switzerland

## Abstract

Host-associated microbiota have been claimed to play a role in hosts’ responses to parasitic infections, often protecting the hosts from infection. We tested for such a role in the crustacean *Daphnia* and the parasitic bacterium *Pasteuria ramosa*, a widely used model system for host-parasite interactions. We first determined the infection phenotype (i.e., resistotype) of eight clonal *D*. *magna* genotypes against four strains of *P*. *ramosa* by attachment test, followed by 16 S rDNA amplicon sequencing to determine if their genotype or their parasite resistotype influences the composition of their microbiome. We then reciprocally transplanted the microbiota of two host genotypes with opposite resistotypes to four *P*. *ramosa* isolates, followed by a reassessment of their resistotype after transplantation. We found significant differences in microbiome composition and structure between *Daphnia* genotypes and between *Daphnia* resistotypes to specific *P*. *ramosa* strains. Reciprocal microbiota exchange or making the *Daphnia* hosts bacteria-free, however, did not influence the resistotypes of the hosts. Thus, in contrary to what has been observed in some taxa, our results suggest that *D*. *magna* susceptibility to *P*. *ramosa* is strongly dictated by the genetic differences of the hosts and is still dependent on *Daphnia*’s first line of immune defense against the esophageal attachment of *P*. *ramosa*, which appears to be uninfluenced by the host’s microbiota.

## Introduction

Until a few years ago, conventional wisdom stated that differences among host populations in resistance to pathogens are largely explained by host genetic differences^1–3^ with environmental factors playing a subordinate role. Recently, microbiota have been added to the list of non-genetic factors that contribute to host resistance against parasites^4–7^. The specificity of infection from a gut pathogen in honeybees, for instance, is purportedly influenced by microbiome composition, and hints at the protective role of the microbiome in counteracting parasite infection^5,8^. Contrastingly, reports that the microbiome can also mediate host susceptibility to diseases have been gaining a lot of attraction in the biomedical sciences^9–11^. The beneficial role of the microbiome to host health has been overtly emphasized in recent years, but their possible influence on host pathogen resistance comes from a handful of investigations from a few host taxa so far. Therefore, this emerging concept requires more evidence before a solid generalization across taxa can be made.

Next generation sequencing has allowed researchers to characterize the microbiome composition of many organisms, resulting in increasingly more complex patterns. Environmental conditions such as diet, can explain variation in microbiome composition between individuals^12,13^, but species-specificity of microbiome composition is still observed, and differences between groups of organisms can still be explained in part by their phylogenetic position^14,15^. Recently, the host genotype in mice was reported to influence gut microbial composition^13,16^, with some members of microbiome being more heritable than others^17^. Moreover, genetic variation in immune-related pathways between human hosts is found to be associated with microbiome composition, lending support to the idea that aside from environmental factors, immune traits can influence microbiome composition^15,18,19^. However, it is not known whether it is the differences in overall host genetic background or the differences in specific immune traits that influences the microbiome composition in a given host taxa. Teasing apart these factors would allow to understand the role that the microbiome plays on immune traits and pathogen resistance. This would be especially interesting among invertebrates that lack adaptive immunity.

We used the *Daphnia-Pasteuria* host-parasite system to address these questions. Genetic variation is observed among natural genotypes of *D*. *magna*, and quantitative trait locus (QTL) approaches have been developed to address questions on the genetic basis of parasite resistance^20,21^. Many microbial parasites infect *Daphnia*, and one of them, *P*. *ramosa*, a gram-positive bacterium of the class *Bacilli*, was shown to display a co-evolutionary arms race with its hosts^22,23^. Furthermore, the role of microbiome on *Daphnia* fitness has been established and the experimental system can be manipulated such that the production of gnotobiotic *Daphnia* is possible^24^. These characteristics make *Daphnia* a good model to disentangle the contribution of host genetic background and microbiome composition, and to determine if the microbiome plays a role in *Daphnia-P*. *ramosa* interactions.

The interaction of *P*. *ramosa* with *D*. *magna* reveals a very high genetic specificity, visible as strong host genotype by parasite genotype interaction with an almost perfect binary infection outcome^25,26^. When a potential host encounters the parasite spores, the spores are activated followed by attachment to the esophagus wall. After successful attachment, the spores penetrate the foregut epithelium into the host body cavity and start proliferating in the haemolymph. Eventually, the host dies and releases *Pasteuria* spores in the environment^27^. Infections are highly virulent with severe reduction in host fecundity and lifespan. Most variation in infection success is explained by the first line of defense, i.e., the ability of the host to prevent attachment to the esophagus, the entry to *Daphnia*’s foregut^25^. Susceptibility to the host’s first line of defense can be assessed with an esophagus attachment test, during which fluorescently labeled *P*. *ramosa* spores are observed while attaching to the esophagus wall of the host. No esophageal attachment indicates resistance to the parasite^25^.

The main goal of this study is to determine if the microbiome plays a role in the resistance of *D*. *magna* to *P*. *ramosa*. We evaluated this by determining the infection phenotype of 8 *Daphnia* genotypes (=clones) to four *P*. *ramosa* strains, followed by comparison of their microbiome structure and composition based on genotype and resistance phenotype (termed resistotype in this study) to four *Pasteuria* strains. We then performed a reciprocal transplant experiment where we swapped the microbiome of resistant and susceptible *D*. *magna* genotypes and tested for resistance to one *P*. *ramosa* strain. Furthermore, we compared the resistotype of these *D*. *magna* genotypes when they were bacteria-free. We found that *D*. *magna* genotypes vary in microbiome composition even after years of culturing them under the same environmental conditions, and that statistical analysis indicated that their microbiome composition can potentially be structured by their resistotype. However, replacing the microbiome of the hosts or making the hosts bacteria-free have not altered their resistance or susceptibility to a *P*. *ramosa* pathogen.

## Results

### Resistotype of *D. magna* genotypes

Four *Pasteuria* strains were tested for attachment in the esophagus to identify *Daphnia* resistotypes (n = 31) (Table 1). Most *Daphnia* genotypes tested were resistant to Pr_C1 and Pr_P20, while half of the genotypes tested were susceptible to Pr_C19 and Pr_P15. Two genotypes were resistant to all four *Pasteuria* pathogens (clones Belgium_1, BE-OHZ-M5 and Germany, DE-K35-Inb1), while the *Daphnia* genotype from Hungary (HU-H0-2) was susceptible to all four pathogens. The Israel (IL-M1-1) clone died in culture and was not tested for Pr_P15 and Pr_P20.

**Table 1:** Infection phenotype (i.e., resistotype) of eight *D*. *magna* genotypes to four *P*. *ramosa* pathogenic strains tested via attachment testing to the esophagus or hindgut.

*Daphnia* ID	Pr_	Pr_	Pr_	Pr_	Resistotype_	Country Origin	Location	Latitude-Longitude	Life Stage
									Collected
	C1	C19	P15	P20	all				
BE-OHZ-M5	R	R	R	R	RRRR	Belgium_1	Oud Heverlee, Belgium	50° 50′ 14′′, 4° 39′ 48′′	Hatched from ephippium
BE-OHZ-M10	S	S	R	R	SSRR	Belgium_2	Oud Heverlee, Belgium	50° 50′ 14”, 4° 39′ 48′′	Hatched from ephippium
QTL-IXF-1	R	R	S	R	RRSR	F1_lab_breed	Basel, Switzerland	47° 33′ 37′′, 7° 34′ 57′′	Hatched from ephippium
FI-Xinb3	R	S	S	R	RSSR	Finland	Tvärminne, Finland	59° 50′ 31′′, 23° 12′ 6′′	Hatched from ephippium
DE-K35-Iinb1	R	R	R	R	RRRR	Germany	Ismaninger Teiche, Germany	48° 12′ 24′′, 11° 42′ 35′′	Hatched from ephippium
HU-HO-2	S	S	S	S	SSSS	Hungary	Kikungsagi-nemzeti Park, Hungary	46° 50′ 14′′, 19° 11′ 41′′	Field-collected individual
IR-GG1-1	R	S	R	R	RSRR	Iran	Lake Guru-göl, Iran	37° 55′ 3.8′′, 46° 42′ 7.8′′	Hatched from ephippium
IL-M1-1	R	R			RR	Israel	Mamilla Pond, Jerusalem	31° 42′ 52′′, 35° 3′ 3.5′′	Hatched from ephippium

### Common microbiome among *D. magna* genotypes

We sequenced the V3 to V5 hypervariable region of the 16 S rDNA of 31 samples belonging to eight *D*. *magna* genotypes including one positive control (mock community) and analyzed 432, 318 sequences after denoising (3893–47570 per sample). Out of 28 genera used in the DNA mock community, 21 were represented at relative abundances similar to the DNA proportions used for sequencing. The use of 97% cut-off in picking Operational Taxonomic Units (OTUs) only gave resolution at the genus or family level identification and not at the strain level (see Supplementary Fig. S1). Strains belonging to *Actinobacteria* were not seen in the DNA mock community sequences but the V3-V5 16 S rDNA primers were able to capture the majority of the bacterial groups. *Actinobacteria* sequences were however seen in 4 (out of 31) *D*. *magna* samples albeit very rare and low in abundances. Qi *et al*.^28^ have seen *Actinobacteria* sequences in their metagenomics survey of *Daphnia* microbiome and found these to be abundant in *D*. *pulicaria* but very rare in *D*. *magna* and *D*. *pulex*. The DNA mock community sequences were not included in the downstream analysis.

**Figure 1:**
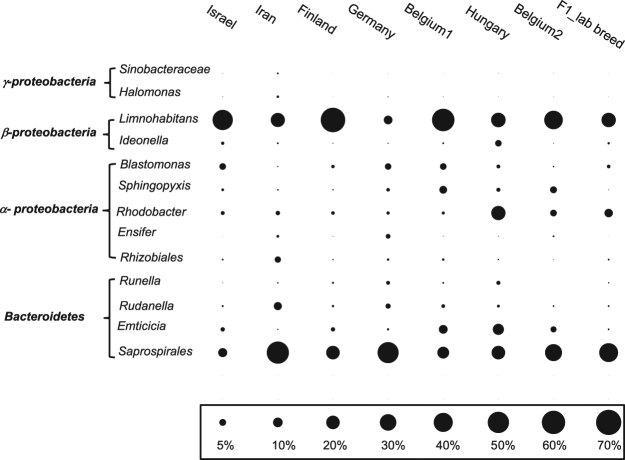
Common microbiome found in 75% of *D*. *magna* samples (n = 31) originated from different populations. These thirteen genera/families also represent the abundant OTUs in the *D*. *magna* microbiome consortium. Bubbles represent OTU counts shown in relative abundance (%).

A total of 6548 OTUs (≥97% ID) were represented in the 31 *D*. *magna* samples but after removing singletons and chloroplast sequences, only 455 OTUs remained to be used for the analysis. Of these OTUs, 13 were present in 23 (out of 35) of the *D*. *magna* samples comprising genera and orders at different relative proportions (Fig. 1). The abundant microbiome populations were dominated by bacteria having homologous 16 S rDNA sequences to *Limnohabitans* (Beta-proteobacteria, 22–66% of the total counts), *Saprospirales* (Bacteroidetes, 9%- 54%), and *Rhodobacter* (Alpha-proteobacteria, 1–22%). Bacterial sequences homologous to *Emticicia*, *Rudanella* and *Runella* (Bacteroidetes, 0.5–3%); *Rhizobiales*, *Ensifer*, *Sphingopyxis* and *Blastomonas* (Alpha-proteobacteria, 0.2–7%); *Ideonella* (Beta-proteobacteria, 0.3–5%); *Halomonas* and *Sinobactereacea* (Gamma-proteobacteria, ≤ 0.6%) were the other least abundant but common microbiome among *D*. *magna*.

Bacterial OTUs that were >1% in relative abundance but were only found in 15 out of 31 *D*. *magna* samples had homologous 16 S rDNA sequences to the Flavobacteriales of the genera *Chryseobacterium* and *Flavobacterium* and the Sphingomonadales of the genera *Sphingomonas* and *Sphingobium*. Some bacteria such as *Hydrogenophaga*, *Caulobacter*, *Myxococcales*, *Pseudomonas*, *Pedobacter and Bacillus* were abundant in some *Daphnia* genotypes (Supplementary Fig. S2).

### Microbiome similarity by genotype and resistotype

**Figure 2:**
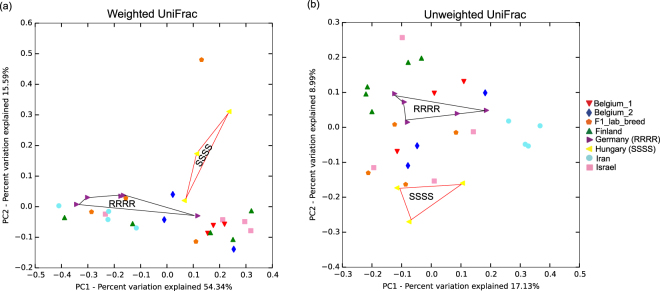
Differences in microbial community structure within and between different *Daphnia* genotypes. Principal Coordinate Analysis based on (**a**) Weighted and (**b**) Unweighted UniFrac Distances that were rarified and jacknifed at equal sequence depth of 3893. Similar symbol represents samples belonging to the same *Daphnia* genotype. Microbial composition differs by *Daphnia* genotypes as shown by ANOSIM (Weighted UniFrac: R = 0.22, p = 0.016; Unweighted UniFrac: R = 0.50, p = 0.001) and ADONIS (Weighted UniFrac: R^2^ = 0.42, p = 0.007; Unweighted UniFrac: ADONIS, R^2^ = 0.39, p = 0.001) statistical analyses with 999 permutations. Polygons show the distinct microbiome between *Daphnia* genotypes with opposite resistotypes to four *Pasteuria* pathogen. Red polygon represents the microbiome of Hungary clone (SSSS) and black polygon represents the microbiome of Germany clone (RRRR).

We plotted the principal coordinates of the Weighted and Unweighted UniFrac distances (Fig. 2a,b) to assess the similarity of the microbiome composition between eight *Daphnia* genotypes. A number of distinct clusters between genotypes were seen, suggesting variation in the microbiome composition between host genotypes. ANOSIM analysis supports statistically significant differences in bacterial community composition between host genotypes (Unweighted UniFrac, ANOSIM R = 0.50, p = 0.001). Similar results were obtained using ADONIS both from Unweighted (R^2^ = 0.39, p = 0.001) and Weighted UniFrac Distances (ANOSIM R = 0.22, p = 0.016; ADONIS: R^2^ = 0.42, p = 0.007) and Bray-Curtis Distance (ANOSIM R = 0.22, p = 0.004; ADONIS: R^2^ = 0.39, p = 0.006). Dissimilarity in the microbiome composition corresponded with the geographic origin of *D*. *magna* genotypes. For instance, the Iran clone showed a more distinct microbiome composition than the Finland clone (Unweighted UniFrac distance = 0.67, p < 0.01), even though both were hatched from resting eggs in the lab. A similar observation was seen between the Germany and the Hungary clones (Unweighted UniFrac distance = 0.58, p = 0.052, black and red polygons, respectively, in Fig. 2a,b). Notably, these two *Daphnia* clones have opposite resistotypes to four *P*. *ramosa* strains. The F1 hybrid clone (F1_lab breed) had a microbial composition similar to its parents, the Finland and Germany clones (Unweighted UniFrac distance = 0.50 and 0.53 respectively, p > 0.05), although this sexual offspring has been hatched from ephippia.

**Figure 3:**
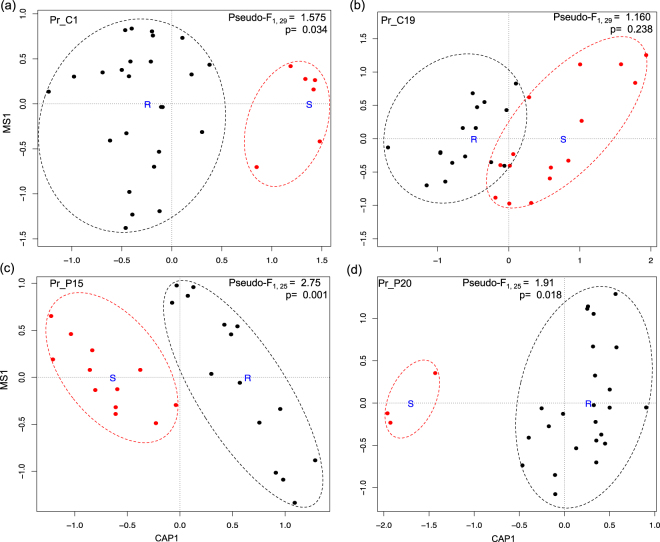
Biplot of db-RDA analysis using Unweighted UniFrac Distance, showing the clustering of microbiome composition between *D*. *magna* samples, with the analysis constrained by their resistotype to each *P*. *ramosa* strain (**a**) Pr_C1, (**b**) Pr_C19, (**c**) Pr_P15, and (**d**) Pr_P20. R (resistant) and S (susceptible) act as centroids to the clusters. Red colored samples belong to the susceptible *D*. *magna* resistotype and black colored samples belong to the resistant *D*. *magna* resistotype for the respective *P*. *ramosa* strains. Pseudo-F and p values < 0.05 denotes statistically significant variation between the microbiome of the *Daphnia* resistotypes.

To further determine if the resistotype of the *D*. *magna* clones against four *P*. *ramosa* strains can explain the variation in the microbiome composition besides the genotypic effect, we assessed the variation of the microbiome composition between *Daphnia* using their resistotype to individual *Pasteuria* strains as the constraining variable employing three distance matrices. Distance-based Redundancy Analyses (db-RDA) based on Unweighted UniFrac distance matrix showed significant clustering of *Daphnia* microbiome by resistotypes to Pr_C1, Pr_P15 and Pr_P20 (p values < 0.05, Fig. 3a,c and d) but not with Pr_C19 (p > 0.05, Fig. 3b). However, db-RDA performed on Weighted UniFrac distance matrix did not show significant clustering in the microbiome composition between *Daphnia* based on resistotypes to Pr_C1, Pr_C19, Pr_P15 and Pr_P20 (Weighted Unifrac: p = 0.07, 0.63, 0.11 and 0.07, respectively; Supplementary Fig. S3A–D). Similarly, db-RDA analysis performed using Bray-Curtis distance matrix also did not show significant clustering in the microbiome composition between *Daphnia* resistotypes to Pr_C1, Pr_C19 and Pr_P15 but showed significant microbiome clustering based on Pr_20 (Bray-Curtis: p = 0.08, 0.56, 0.07 and 0.05, respectively; Supplementary Fig. S4A–D). The analyses suggest that the microbiome composition between *D*. *magna* could potentially be influenced by their resistotype to specific *P*. *ramosa* strains.

### Microbiome composition of highly-resistant and highly-susceptible Daphnia

**Figure 4:**
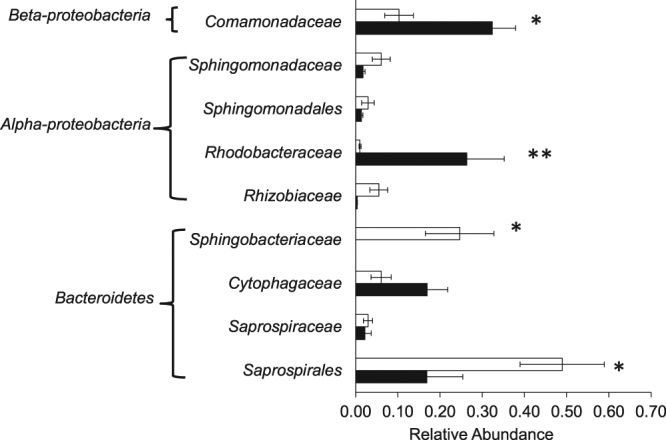
Differences in microbiome composition between highly-resistant (RRRR) *D*. *magna* genotype from Germany (DE-K35-Inb1) and highly-susceptible (SSSS) *D*. *magna* genotype from Hungary (HU-HO-2) shown as average relative abundance at the family level. Statistical comparison was performed using Student’s T-test with levels of significance denoted by *for p < 0.05 and ** for p < 0.01.

We compared the alpha diversity and bacterial composition of the highly-resistant (Germany, RRRR) and highly-susceptible *Daphnia* (Hungary, SSSS) to gain an insight on the differences of the microbiome composition between the two extreme *Daphnia* resistotypes. These clones were used in the reverse microbiome transplant experiment described below. *Daphnia* genotype from Germany is significantly less species rich than the Hungary genotype based on Chao1 and number of observed OTUs metrics (Students T-test; p = 0.03 and 0.03, respectively). In terms of bacterial abundance, the highly-resistant German genotype contains a much higher proportion of bacteria from the family *Saprospirales* (p = 0.05), *Sphingomonadaceae*, *Sphingobacteriaceae* (p = 0.05) and *Rhizobiaceae* while it has a significantly lower proportion of bacteria from *Comamonadaceae* (p = 0.01), *Rhodobacteraceae* (p = 0.008) and *Cytophagaceae* (p = 0.06) (Fig. 4) than the highly-susceptible Hungarian genotype.

### Microbiota swapping between *D. magna* of opposing resistotypes

We used Hungarian (SSSS) and German (RRRR) genotypes for the reverse microbiota transplant experiment to determine if microbiota swapping will change the original resistotype of the respective *Daphnia* clone. *Pasteuria* attachment tests showed that Pr_C1 attached to the esophagus wall of all Hungarian genotype replicates, regardless if the animal is bacteria-free, if it contains its native microbiota or if the *Pasteuria*-susceptible *Daphnia* contains the microbiota of a *Pasteuria*-resistant German genotype (Table 2). Similarly, *Pasteuria* Pr_C1 failed to attach on the esophagus wall of all Germany genotype replicates in all treatments. This suggests that the respective *Daphnia* genotypes retained their infection phenotype regardless of their microbiota or even in the absence of microbiota.

**Table 2:** Results of the infection phenotyping of bacteria-free animals and reciprocally transplanted microbiota between highly resistant *Daphnia* from Germany and highly-susceptible *Daphnia* from Hungary.

Treatment	Positive attachment
**A**. **Pr_C1 Susceptible** ***Daphnia*** **(SSSS**, **Hungary clone)**	
BacFree (n = 20)	100%
Susceptible *Daphnia* + own microbiota (n = 17)	100%
Susceptible *Daphnia* + microbiota of Resistant *Daphnia* (n = 18)	100%
**B**. **Pr_C1 Resistant** ***Daphnia*** **(RRRR**, **Germany clone)**	
BacFree (n = 7)	0%
Resistant *Daphnia* + own microbiota (n = 8)	0%
Resistant *Daphnia* + microbiota of Susceptible *Daphnia* (n = 14)	0%

## Discussion

Genetics and microbiota can have important functions in health and performance of animals. Here we report that genotypes of the model system *D*. *magna* from various geographic backgrounds still differed in their microbiome composition even after years of culturing the animals under the same dietary and environmental growth conditions. This indicates that the effects of the host genotype and of the host microbiota on the expression of phenotypic traits can be confounded. Both effects can be very important in the ecology and evolution of the system, but in different ways. For example, genes are only transmitted vertically, while microbiota can be transmitted vertically and horizontally. During migration, microbiota may be lost and in new habitats, new members may be acquired. To understand the expression and evolution of phenotypic traits, it is therefore necessary to disentangle host genetics from microbiota effects. Here we tested whether the first line of defense (i.e., the attachment of parasite in the esophagus or foregut entry) characterized by resistotypes of the water flea *D*. *magna*, are influenced by host genetic differences, or by the host’s microbiota as has been suggested in other systems.

We found that genotypic variation and parasite susceptibility and resistance of *D*. *magna* to specific *P*. *ramosa* strains, can correlate with differences in the microbial composition of the daphnid hosts (see Figs 2–3). In humans and mice, host genetics is reported to be a factor in shaping the composition and diversity of the microbiome^17,29^ and the abundance of some members of microbiota are associated with immune-related genes^18,30,31^. However, despite significant variation in the microbiome composition and structure between daphnid hosts (especially between two highly-opposite resistotypes), we have not seen a microbiota influence on the resistance trait of *D*. *magna* against the parasite *P*. *ramosa* (at least with Pr_C1 strain) after performing a reciprocal microbiome exchange. Remarkably, the resistotypes of the two *Daphnia* genotypes to the parasite were also not altered even when the animals were in a bacteria-free state, which strongly supports our conclusion, that the microbiome may not play a role in the innate immunity (first line of defense) of *D*. *magna* against a *P*. *ramosa* pathogen. These results are in direct contrast from what is reported in other taxa such as in bumblebees where it has been shown that gut microbiome plays a protective role against trypanosomatid parasites^5^, with the host genotype playing a secondary role in their immunity^8^, and in wild *Anopheles gambiae* where a member of the midgut microbiome renders the insect resistant to the malaria-causing *Plasmodium*^32^. In a marine crustacean *Palaemon macrodactylus*, a bacterium constantly isolated from the eggs can protect the shrimp embryo from a fungal pathogen by releasing an anti-fungal chemical^33^. In human disease-related pathogenesis, a fecal microbiota probiotic could combat infection of the pathogen *Clostridium difficile* to alleviate suffering from chronic intestinal bowel disorder^34,35^. Furthermore, the human gut microbiota member, *Bacteroides fragilis*, has been proposed as a potential probiotic to improve autistic behavioral symptoms^36^. The disparity between our study and the emerging paradigm that the microbiome plays a major role in the resistance against parasite infection or disease pathogenesis emphasizes the idea that the microbiome’s role in immunity could be organism-dependent (or at least parasite-dependent) and should not be generalized across all taxa. Notably, the *Daphnia*-*Pasteuria* system exhibits strong host-parasite species interaction, with host infection rate dependent on the presence of diverse parasite strains that would specifically infect matching host genotypes^37^. The co-evolutionary arms race between *Daphnia* and *Pasteuria* may be a stronger driving factor on the evolution of innate immunity in *D*. *magna* against the pathogen and could mask the influence of microbiota (if there is any). Moreover, the difference of the *Daphnia*-*Pasteuria* system compared to other host-parasite systems may lie with how the *Pasteuria* infection process takes place, the decisive step being the attachment of *Pasteuria* to the esophagus. *P*. *ramosa* can only infect *Daphnia* if it manages to attach to the esophagus of the daphnid host^25^. It is evident that this inhibition of attachment is accomplished by the epithelial immunity or barrier defenses present in the esophagus (the esophagus being the extension of the foregut) of resistant *Daphnia*, and appears to be entirely independent from the microbiome influence. Interestingly, the absence of microbiome in resistant *Daphnia* did not alter the barrier defenses. Bacteria-free organisms are known to have abnormal and immature gut development^38–40^ which impairs mucosal immunity in the gut leading to disease susceptibility. In tse-tse flies, microbiota-free organisms are susceptible to trypanosome infection because of a compromised peritoneal matrix that act as a physical barrier between lumen and epithelial cells in their midgut^6^.

In this study, we only evaluated the influence of the microbiome on *D*. *magna*’s first line of defense against pathogen infection, which is the prevention of infection via resistance against gut entry. Microbiota may possibly play a role on *Daphnia*’s second line of defense, which is the prevention of pathogen growth inside the body or infection tolerance by reducing the damage of the infection through fitness compensation. Indeed, *Pasteuria* susceptible *Daphnia* can mount an immune response by increasing the number of phagocytes in its haemolymph when infected^41^, or by maintaining similar levels of fecundity before total sterilization^42^. To investigate the possible role of the microbiome on *Daphnia*’s second line of defense, a *Pasteuria* susceptible *Daphnia* transplanted with various microbiome members could be evaluated against pathogen growth followed by fitness measurement in the presence of infection.

Because *Daphnia* is being used as a model system for environmental genomics^43^, exploring the possible contribution of the microbiome on the adaptive response of *Daphnia* to changing environments could be interesting. We have previously shown that the microbiome has a significant effect on *Daphnia* fitness^24^ and there is a significant variation in the microbiome composition between *Daphnia* genotypes (in this study). Host adaptation to changing environmental conditions could shape their microbiome composition and could confer differences in fitness between *Daphnia* genotypes. The genetic basis of the differing tolerance of natural populations of *Daphnia* to high copper concentrations^44,45^ and temperature^46^ has been investigated previously. The interaction of host genetics and the microbiome in this context could be an interesting area for further investigation.

## Conclusion

This is the first study to show that despite considerable differences in the microbiome composition between *Daphnia* genotypes, particularly that of *P*. *ramosa*-susceptible and *P*. *ramosa*-resistant *Daphnia*, reciprocal swapping of their microbiota did not alter the susceptibility or resistance of the respective daphnid hosts against a *Pasteuria* pathogen, as demonstrated by attachment test of the parasite in the host’s esophagus. More importantly, despite the known contribution of the microbiome to host immunity, a *Pasteuria*-resistant *Daphnia* that was raised bacteria-free was able to maintain its barrier defenses against parasite entry, suggesting that the microbiome does not influence the first line of defense in *Daphnia*. Further investigation of the microbiome’s role on host immunity could be focused on the second line of defense.

## Methods

### Animals

Eight *D*. *magna* genotypes were collected from different geographic regions, either as planktonic individuals or as resting eggs which were subsequently hatched and kept as clonal lines for several years (Table 1). Two clones included are the parents of a standing QTL panel^47,20^, one of which (FI-Xinb3) was selfed three times, while the other (DE-K35-Iinb1) was selfed once. The F1_lab_breed (QTL-IXF-1) genotype is the sexual offspring of these two parent clones and was hatched from a resting egg in the lab^47^. Animals were maintained in artificial *Daphnia* medium (ADaM^48^, for at least 20 generations under standardized environmental conditions; 20 °C, 16:8 light dark photoperiod, the green algae *Scenedesmus* sp. as only food and animals cultured.

Only female adults were sampled for the 16 S rDNA profiling. Five jars with 80 ml ADaM containing two *Daphnia* of each genotype were grown for 4 weeks under standard laboratory conditions fed with 10 million cells algae per jar every other day. ADaM was replaced every week and only 2 adult individuals were kept in the jar every ADaM change. Animals were washed twice in sterile ADaM to remove unassociated bacteria before storage in −20 °C prior to DNA extraction.

### Resistotype determination via esophagus-attachment test

Different *D*. *magna* genotypes were attachment-tested against four *Pasteuria* clones/isolates as described by Duneau *et al*.^25^ *Daphnia* show a binary infection outcome to *Pasteuria* pathogens; susceptible animals allow *P*. *ramosa* attachment to the esophagus or to the hindgut while resistant animals display no sign of attachment. *P*. *ramosa* Pr_C1 and Pr_C19 clones were isolated from infected *D*. *magna* from Moscow (Russia) and Gaarzerfeld (Germany), respectively, and previously characterized in Luijckx *et al*.^26^. Pr_C1 and Pr_C19 mode of attachment is through the esophagus. *P*. *ramosa* Pr_P15 and Pr_P20 are isolates that have been propagated in the same *D*. *magna* host clones several times, but where not cloned. Pr_P20 was isolated from an infected *D*. *magna* female that was collected in 2011 from Aegelsee, Switzerland. Pr_P20 attaches to the host in the esophagus. Pr_P15 is derived from an infected *D*. *magna* collected in Belgium and is unique in that it attaches usually to the hindgut of the host.

*P*. *ramosa* spores were fluorescently labeled and attachment tests performed as described by Duneau *et al*.^25^. Briefly, spores were labeled with fluorescein-5(6)-isothiocyanate (FITC) dye at a concentration of 1 mg in 0.1 M NaCO_3_ (pH 9.1). The solution was kept in the dark for 2 hours and washed 3–5 times via centrifugation. *Daphnia* were exposed to resuspended spores (5000 spores per individual) for 1 hour in the dark. Animals were then examined for spore attachment with an inverted epifluorescent microscope (Leica DM 2500) at 400× magnification. Resistotypes were defined as 4 letter codes (e.g. RSSR) composed of R (for resistance, no attachment) and S (susceptibility, attachment) for *P*. *ramosa* strains Pr_C1, Pr_C19, Pr_P15, Pr_P20, respectively.

### DNA extraction

Frozen animal tissues were bead-beat with two pieces of acid-washed and autoclaved 1.0 mm beads and a 100 µl of 0.1-mm silica zirconian beads in PCR water in a FastPrep F120 cell disruptor (Qbiogene, USA) for 30 s at 4 m·s^−1^. DNA extraction was continued following the steps described in the DNeasy Blood and Tissue Kit (Qiagen, Netherlands). PCR water was used as a control for the extraction procedure and went through the same extraction and PCR amplification steps as the *Daphnia* samples.

### 454 Barcode Tagging

Two PCR reactions steps were carried out in amplifying the V3-V5 hypervariable region of the 16 S rDNA with Roche 454 barcodes. The first PCR amplification step involved using the primer pairs F (5′-ACACGGYCCARACTCCTAC-3′) and R (5′-TTGCWTCGAATTAAWCCAC-3′) that targets the 327–969 bp of the 16 S rDNA using Phusion High Fidelity Taq (New England Biolab, USA) with this PCR program: 98 °C for 1 min, 15 cycles of 98 °C for 10 s, 50 °C for 20 s and 72 °C for 30 s with a final extension time of 72 °C for 5 min. PCR reactions were treated with ExoSap (Fermentas, USA), to remove unincorporated nucleotides and primers. A second PCR reaction step was prepared using the first PCR purified product as DNA template and re-amplified with F/R primer pairs containing the Libl fusion sequence and barcodes (PFx: 5′-CCATCTCATCCCTGCGTGTCTCCGACTCAGbarcodeF primer-3′.

PRx: 5′-CCTATCCCCTGTGTGCCTTGGCAGTCTCAGR primer-3′) using the same PCR program and cycles. Amplified products were purified using Agencourt Ampure XP beads (Beckman and Coulter, USA) and eluted in sterile TE buffer. DNA concentrations were measured using Qubit 2.0 Fluorometer (Invitrogen, USA) and a pool of equimolar amounts (30 ng/µL) of barcoded products were sequenced on 454 GS-FLX instrument with LibL Titanium chemistry (Roche, USA) at Microsynth (Balgach, Switzerland). A DNA mock community consisting of 76 bacterial strains belonging to 28 genera and families of various phyla was also tagged with a barcode and was sequenced as a positive control (see Supplementary Table S1). The mock community consisted of bacterial isolates previously isolated from *Daphnia* animals either freshly collected from the field or maintained in the lab, the algal food cultures (*Scenedesmus* sp.) or ADaM (*Daphnia* culture medium). Isolates were chosen to represent different bacterial phylogenies and based on the availability of bacterial cultures.

### Sequence Data Processing and Analysis

Demultiplexed samples were denoised and pre-clustered at 99% level similarity using the method described by Reeder and Knight^49^. Samples were checked for chimeras, low quality sequence and short sequence reads (<150 bp) prior to post-analysis with the Quantitative Insights Into Microbial Ecology (QIIME 1.9.1^50^). OTUs were clustered at 97% sequence similarity with UClust^51^. Representative sequences were picked for taxonomic identification based on Blast 2.2.22^52^. OTU representative sequences were aligned with Pynast^53^, while gaps in the alignment were filtered using the default 16 S rDNA Greengenes alignment lane mask before making the phylogenetic tree using FastTree 2.1.3^54^. Singletons and chloroplast sequences were filtered out of the OTU table before downstream analyses.

### Microbial community composition analysis

To control for sequencing effort, samples were rarified at a minimum of 10 sequences and a maximum depth of 3893 sequences in steps of 50, and performing 20 iterations for each step. The microbial diversity for each *D*. *magna* sample was assessed using 4 alpha diversity indices (Observed_OTUs, Phylogenetic Diversity, Chao1 and Shannon index) at 3893 sequence depth and statistically compared using Student’s T-tests in JMP 12.0 (Cary, NC, USA). Since our main objective is to compare the microbial community between genotypes with contrasting resistotypes for the reciprocal transplant experiment, only alpha diversity of RRRR (Germany, n = 5) and SSSS (Hungary, n = 3) host genotypes were reported in this study.

For microbial composition assessment of *Daphnia* genotypes and resistotypes, the OTU table was rarified at an equal depth of 3893 sequences to generate pairwise distance matrices. Weighted and Unweighted UniFrac and Bray-Curtis distance matrices derived from this rarified beta diversities were used to statistically test for differences in microbial composition using ANOSIM and ADONIS methods with 999 permutations. Distance-based redundancy analysis (db-RDA) was also applied to determine the clustering of *D*. *magna* microbiome composition when constrained by their resistotype to each *P*. *ramosa* strain.

### Reciprocal transplant of microbiota between susceptible and resistant Daphnia

To determine if microbiota influences the resistotype of *D*. *magna* to *Pasteuria* infection, we removed the bacteria, or swapped the microbiota of two *Daphnia* genotypes that were previously tested to be highly-susceptible (SSSS, Hungary clone) and highly-resistant (RRRR, Germany clone) to four *P*. *ramosa* clones and isolates (see Table 1). The detailed methods for synchronizing the animals, the production of bacteria-free *Daphnia* from asexual eggs, the preparation of bacterial suspension, preparation of axenic *Scenedesmus obliquus* algae and subsequent PCR screening for bacteria-free *Daphnia* and algae were previously described in Sison-Mangus *et al*.^24^. Briefly, eggs from 100 individuals of synchronized Hungarian and German *Daphnia* mothers were extracted from the mother’s brood pouch and washed with sterile ADaM three times. Eggs were treated with freshly made Ampicillin (1 mg/mL) and Kanamycin (100 μg/mL) for 48 hours until development of hatchlings. Sterile ADaM and antibiotics were replaced each day. The bacteria-free state of the hatchlings after antibiotics treatment was verified by 16 S rDNA PCR amplification.

Two independent reciprocal transplant experiments were performed using the Hungarian (SSSS) and the German (RRRR) clones as respective experimental animals. After antibiotics removal, bacteria-free *Daphnia* hatchlings were divided into groups representing 3 treatments: 1) Bacteria-free *Daphnia*, 2) *Daphnia* inoculated with microbiota from the same clone, and 3) *Daphnia* inoculated with microbiota coming from clone with opposite resistotype (e.g., German hatchlings received the microbiota from Hungarian clone while the Hungarian hatchlings received the microbiota from German clone). Bacterial suspensions were harvested from the respective *Daphnia* clone sources following the method of Sison-Mangus *et al*.^24^ with 1 ml bacterial suspension given to respective hatchlings from each group. The optical density (OD_600_) of the bacterial suspension ranged from 0.72–0.73. Hatchlings were exposed to bacteria for 24 hours and subsequently washed once before placing in individual experimental jars with 25 million axenic algal food cells.

Hatchlings were grown under the same environmental conditions as above and maintained daily as in Sison-Mangus *et al*.^24^. *P*. *ramosa* Pr_C1 stick testing was done at Day 6 post-hatching using the method described above, but with antibiotic treated spores. For this, the FITC-dyed *P*. *ramosa* spores were treated with triple antibiotic solution (Ampicillin (2 mg/mL), Kanamycin (1 mg/mL) and Tetracyclin (100 μg/mL) for 36 hours in the dark prior to stick testing. This is to prevent unwanted bacteria that maybe present from *Pasteuria* spore solution from contaminating the experimental treatments during the attachment test. Preliminary test showed that *P*. *ramosa* C1 spores remain viable (able to attach to the esophagus of *Daphnia*) after being treated with antibiotics.

### Data availability

The raw sequencing reads have been deposited at the NCBI Short Read Archive under BioProject ID PRJNA407858 with the following BioSample accession numbers: SAMN07665251 to SAMN07665254 for IR-GG1–1 clone, SAMN07665255 to SAMN076652588 for IL-M1-1 clone, SAMN07665259 to SAMN07665261 for HU-HO-2 clone, SAMN07665262 to SAMN07665264 for BE-OHZ-M5 clone, SAMN07665265 to SAMN07665267 for BE-OHZ-M10 clone, SAMN07665268 to SAMN07665272 for FI-Xinb3 clone, SAMN07665273 to SAMN07665277 for DE-K35-Iinb1 clone, SAMN07665278 to SAMN07665281 for QTL-IXF-1 clone and SAMN07695326 for Positive Control.

## Electronic supplementary material

Supplementary Materials
