# Supplementary material for: Host genotype-specific microbiota do not influence the susceptibility of D. magna to a bacterial pathogen
Source: Sci Rep. 2018 Jun 20;8:9407. doi: 10.1038/s41598-018-27681-x (PMC6010447; doi:10.1038/s41598-018-27681-x)

Title: Host genotype-specific microbiota do not influence the susceptibility of *D. magna* to a bacterial pathogen

*Marilou P. Sison-Mangus[1, *], César M. J. A. Metzger [2, 3] and Dieter Ebert [2]*

Supplementary Table S1. Cultured bacterial strains used in DNA mock community sequencing.

**Phylum**	**Order**	**Family**	**Strain name**	**[*]Source**
Actinobacteria	*Actinomycetales*	*Gordoniaceae*	*Gordonia01*	[@]Daphnia_Field
Actinobacteria	*Actinomycetales*	*Micrococcaceae*	*Kocuria02b*	Daphnia_Field
Actinobacteria	*Actinomycetales*	*Micrococcaceae*	*Micrococcus01c*	Daphnia_Field
Actinobacteria	*Actinomycetales*	*Nocardiaceae*	*Rhodococcus01*	Daphnia_Field
Alpha-proteobacteria	*Sphingomonadales*	*Sphingomonadaceae*	*Sphingobium01a*	[$]ADaM
Alpha-proteobacteria	*Sphingomonadales*	*Sphingomonadaceae*	*Sphingobium02a*	ADaM
Alpha-proteobacteria	*Sphingomonadales*	*Sphingomonadaceae*	*Sphingomonas01b*	ADaM
Alpha-proteobacteria	*Sphingomonadales*	*Sphingomonadaceae*	*Sphingomonas02b*	[#]Algae
Alpha-proteobacteria	*Sphingomonadales*	*Sphingomonadaceae*	*Sphingomonas03*	ADaM
Alpha-proteobacteria	*Sphingomonadales*	*Sphingomonadaceae*	*Sphingomonas04*	ADaM
Alpha-proteobacteria	*Sphingomonadales*	*Sphingomonadaceae*	*Sphingomonas05*	ADaM
Alpha-proteobacteria	*Sphingomonadales*	*Sphingomonadaceae*	*Sphingomonas06a*	Algae
Alpha-proteobacteria	*Sphingomonadales*	*Sphingomonadaceae*	*Sphingomonas07*	ADaM
Alpha-proteobacteria	*Sphingomonadales*	*Sphingomonadaceae*	*Sphingomonas08*	ADaM
Alpha-proteobacteria	*Sphingomonadales*	*Sphingomonadaceae*	*Sphingopyxis01*	[!]Daphnia_Lab
Alphaproteobacteria	*Caulobacterales*	*Caulobacteraceae*	*Brevundimonas01b*	ADaM
Alphaproteobacteria	*Caulobacterales*	*Caulobacteraceae*	*Brevundimonas02*	Algae
Alphaproteobacteria	*Caulobacterales*	*Caulobacteraceae*	*Brevundimonas01c*	ADaM
Alphaproteobacteria	*Caulobacterales*	*Caulobacteraceae*	*Brevundimonas04*	ADaM
Alphaproteobacteria	*Caulobacterales*	*Caulobacteraceae*	*Brevundimonas05*	ADaM
Alphaproteobacteria	*Caulobacterales*	*Caulobacteraceae*	*Brevundimonas06*	ADaM
Alphaproteobacteria	*Caulobacterales*	*Caulobacteraceae*	*Brevundimonas01d*	Algae
Alphaproteobacteria	*Caulobacterales*	*Caulobacteraceae*	*Brevundimonas08*	ADaM
Alphaproteobacteria	*Rhizobiales*	*Rhizobiaceae*	*Rhizobium01*	ADaM
Alphaproteobacteria	*Rhizobiales*	*Rhizobiaceae*	*Rhizobium02*	Algae
Alphaproteobacteria	*Rhizobiales*	*Rhizobiaceae*	*Rhizobium03*	Daphnia_Field
Bacteroidetes	*Flavobacteriales*	*[Weeksellaceae]*	*Chryseobacterium01*	Daphnia_Field
Bacteroidetes	*Flavobacteriales*	*Flavobacteriaceae*	*Flavobacterium01*	Daphnia_Field
Beta-proteobacteria	*Burkholderiales*		*Burkholderiales01*	ADaM

Phylum	Order	Family	Strain name	*Source
Beta-proteobacteria	*Burkholderiales*		*Burkholderiales02*	ADaM
Beta-proteobacteria	*Burkholderiales*	*Comamonadaceae*	*Hydrogenophaga01*	Daphnia_Field
Beta-proteobacteria	*Burkholderiales*	*Comamonadaceae*	*Hydrogenophaga02*	Daphnia_Lab
Beta-proteobacteria	*Burkholderiales*	*Burkholderiaceae*	*Limnobacter01*	Daphnia_Field
Beta-proteobacteria	*Burkholderiales*	*Oxalobacteraceae*	*Massilia01*	ADaM
Beta-proteobacteria	*Burkholderiales*		*Burkholderiales02*	ADaM
Beta-proteobacteria	*Burkholderiales*	*Comamonadaceae*	*Hydrogenophaga01*	Daphnia_Field
Beta-proteobacteria	*Burkholderiales*	*Comamonadaceae*	*Hydrogenophaga02*	Daphnia_Lab
Beta-proteobacteria	*Burkholderiales*	*Burkholderiaceae*	*Limnobacter01*	Daphnia_Field
Beta-proteobacteria	*Burkholderiales*	*Oxalobacteraceae*	*Massilia01*	ADaM
Beta-proteobacteria	*Burkholderiales*	*Oxalobacteraceae*	*Massilia02*	ADaM
Beta-proteobacteria	*Burkholderiales*	*Oxalobacteraceae*	*Massilia03*	ADaM
Beta-proteobacteria	*Burkholderiales*	*Oxalobacteraceae*	*Ralstonia01a*	Algae
Beta-proteobacteria	*Enterobacteriales*	*Enterobacteriaceae*	*Serratia01*	Daphnia_Field
Beta-proteobacteria	*Burkholderiales*	*Comamonadaceae*	*Variovorax01a*	Algae
Beta-proteobacteria	*Burkholderiales*	*Comamonadaceae*	*Variovorax01b*	Algae
Firmicutes	*Bacillales*	*Bacillaceae*	*Bacillus01*	Algae
Firmicutes	*Bacillales*	*Bacillaceae*	*Bacillus02b*	Algae
Firmicutes	*Bacillales*	*Planococcaceae*	*Bacillus03b*	Daphnia_Field
Firmicutes	*Bacillales*	*Planococcaceae*	*Bacillus05b*	Daphnia_Field
Firmicutes	*Bacillales*	*Planococcaceae*	*Bacillus07*	Daphnia_Field
Firmicutes	*Bacillales*	*[Exiguobacteraceae]*	*Exiguobacterium02*	Algae
Gamma-proteobacteria	*Pseudomonadales*	*Moraxellaceae*	*Acinetobacter01*	Algae
Gamma-proteobacteria	*Pseudomonadales*	*Moraxellaceae*	*Acinetobacter02*	ADaM
Gamma-proteobacteria	*Pseudomonadales*	*Pseudomonadaceae*	*Aeromonas02*	Daphnia_Field
Gamma-proteobacteria	*Pseudomonadales*	*Pseudomonadaceae*	*Aeromonas03*	Daphnia_Lab
Gamma-proteobacteria	*Pseudomonadales*	*Pseudomonadaceae*	*Aeromonas03a*	Daphnia_Field
Gamma-proteobacteria	*Pseudomonadales*	*Pseudomonadaceae*	*Aeromonas03b*	Daphnia_Field
Gamma-proteobacteria	*Xanthomonadales*	*Xanthomonadaceae*	*Pseudoxanthomonas01a*	Algae
Gamma-proteobacteria	*Bacillales*	*Staphylococcaceae*	*Staphylococcus01*	Algae
Gamma-proteobacteria	*Bacillales*	*Staphylococcaceae*	*Staphylococcus02*	Daphnia_Field

Phylum	Order	Family	Strain name	*Source
Gamma-proteobacteria	*Xanthomonadales*	*Xanthomonadaceae*	*Xanthomonadaceae01*	Daphnia_Field
Gamma-proteobacteria	*Pseudomonadales*	*Moraxellaceae*	*Enhydrobacter01b*	ADaM
Gamma-proteobacteria	*Pseudomonadales*	*Pseudomonadaceae*	*Pseudomonas01*	ADaM
Gamma-proteobacteria	*Pseudomonadales*	*Pseudomonadaceae*	*Pseudomonas01a*	ADaM
Gamma-proteobacteria	*Pseudomonadales*	*Pseudomonadaceae*	*Pseudomonas02a*	Daphnia_Field
Gamma-proteobacteria	*Pseudomonadales*	*Pseudomonadaceae*	*Pseudomonas02b*	Daphnia_Field
Gamma-proteobacteria	*Pseudomonadales*	*Pseudomonadaceae*	*Pseudomonas02c*	Algae
Gamma-proteobacteria	*Pseudomonadales*	*Pseudomonadaceae*	*Pseudomonas02d*	Algae
Gamma-proteobacteria	*Pseudomonadales*	*Pseudomonadaceae*	*Pseudomonas03a*	ADaM
Gamma-proteobacteria	*Pseudomonadales*	*Pseudomonadaceae*	*Pseudomonas03b*	ADaM
Gamma-proteobacteria	*Pseudomonadales*	*Pseudomonadaceae*	*Pseudomonas04a*	ADaM
Gamma-proteobacteria	*Pseudomonadales*	*Pseudomonadaceae*	*Pseudomonas04b*	ADaM
Gamma-proteobacteria	*Pseudomonadales*	*Pseudomonadaceae*	*Pseudomonas06c*	Daphnia_Field
Gamma-proteobacteria	*Pseudomonadales*	*Pseudomonadaceae*	*Pseudomonas06d*	Daphnia_Field
Gamma-proteobacteria	*Pseudomonadales*	*Pseudomonadaceae*	*Pseudomonas07b*	Daphnia_Field
Gamma-proteobacteria	*Pseudomonadales*	*Pseudomonadaceae*	*Pseudomonas07c*	Daphnia_Field
Gamma-proteobacteria	*Pseudomonadales*	*Pseudomonadaceae*	*Pseudomonas10a*	Daphnia_Field
Gamma-proteobacteria	*Pseudomonadales*	*Pseudomonadaceae*	*Pseudomonas10b*	Daphnia_Field
Gamma-proteobacteria	*Pseudomonadales*	*Pseudomonadaceae*	*Pseudomonas12*	Daphnia_Field
Gamma-proteobacteria	*Pseudomonadales*	*Pseudomonadaceae*	*Pseudomonas15*	Daphnia_Field
Thermi	*Deinococcales*	*Deinococcaceae*	*Deinococcus02*	Algae

[*]Bacteria where isolated from the following sources:
[@]Daphnia_Field- animal freshly collected from freshwater pond in Aegelsee, CH
[!]Daphnia_Lab – cultured animal maintained in ADaM in the lab
[$]ADaM – culture medium for growing *Daphnia*
[#]Algae – algal food (*Scenedesmus* sp.) culture

Supplementary Fig. S1. Proportions of mock DNA bacterial community and the resulting 16s rDNA sequence after 454 sequencing. All, except Actinobacteria strains and 2 Burkholderiales strains, showed up in the sequencing of the mock community.

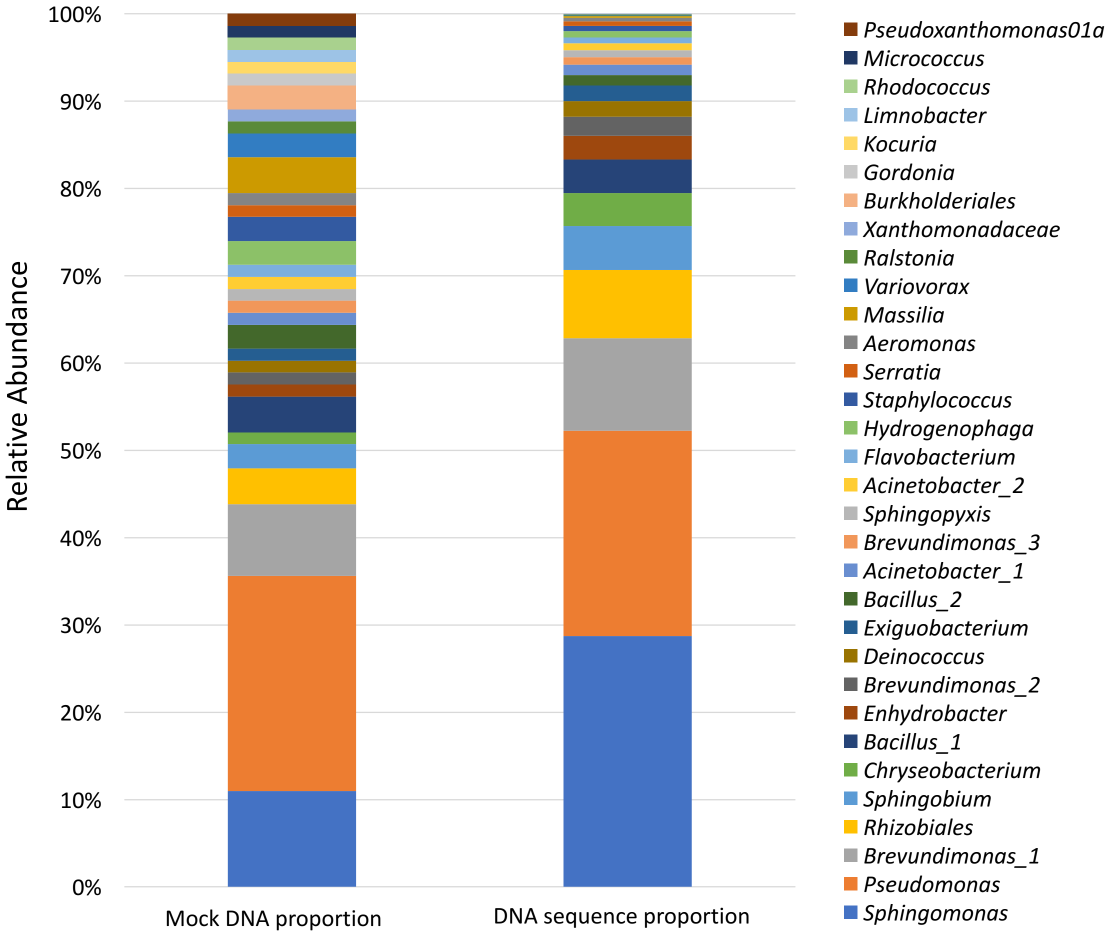

Supplementary Fig. S2. Relative abundance of microbiome present in 8 *Daphnia* genotypes (n= 31) at the genus/family taxonomic level.

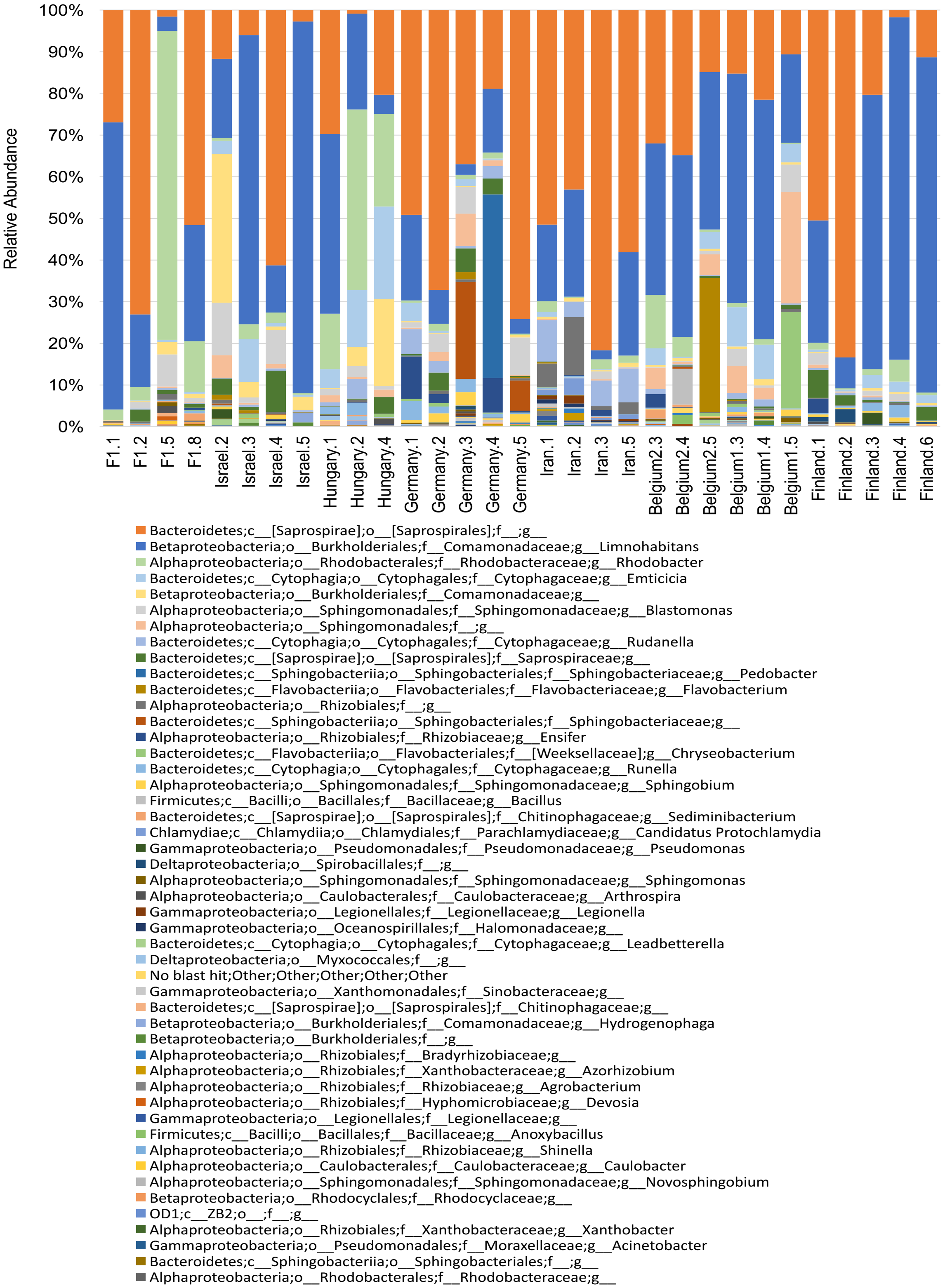

Supplementary Fig. S3. Biplot of db-RDA analysis using Weighted UniFrac Distance, showing the clustering of microbiome composition between *D. magna* samples, with the analysis constrained by their resistotype to each *P. ramosa* strain a) Pr_C1, b) Pr_C19, c) Pr_P15, and d) Pr_P20. R (resistant) and S (susceptible) act as centroids to the clusters. Red colored samples belong to the susceptible resistotype and black colored samples belong to the resistant resistotype for the respective *P. ramosa* strains. Pseudo-F and p values < 0.05 denotes statistically significant variation between the microbiome of the *Daphnia* resistotypes.

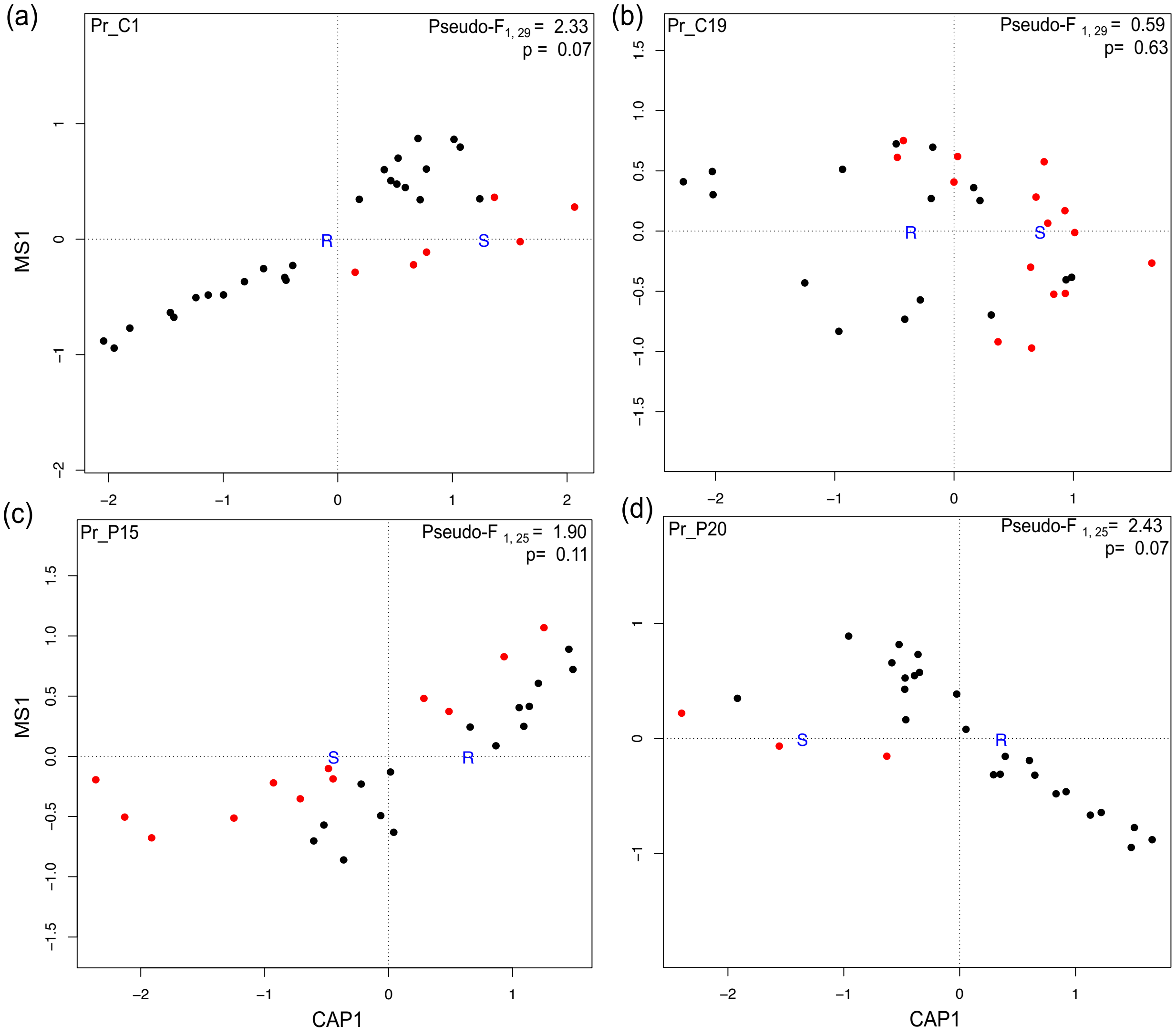

Supplementary Fig. S4. Biplot of db-RDA analysis using Bray-Curtis Distance, showing the clustering of microbiome composition between *D. magna* samples, with the analysis constrained by their resistotype to each *P. ramosa* strain a) Pr_C1, b) Pr_C19, c) Pr_P15, and d) Pr_P20. R (resistant) and S (susceptible) act as centroids to the clusters. Red colored samples belong to the susceptible resistotype and black colored samples belong to the resistant resistotype for the respective *P. ramosa* strains. Pseudo-F and p values < 0.05 denotes statistically significant variation between the microbiome of the *Daphnia* resistotypes.

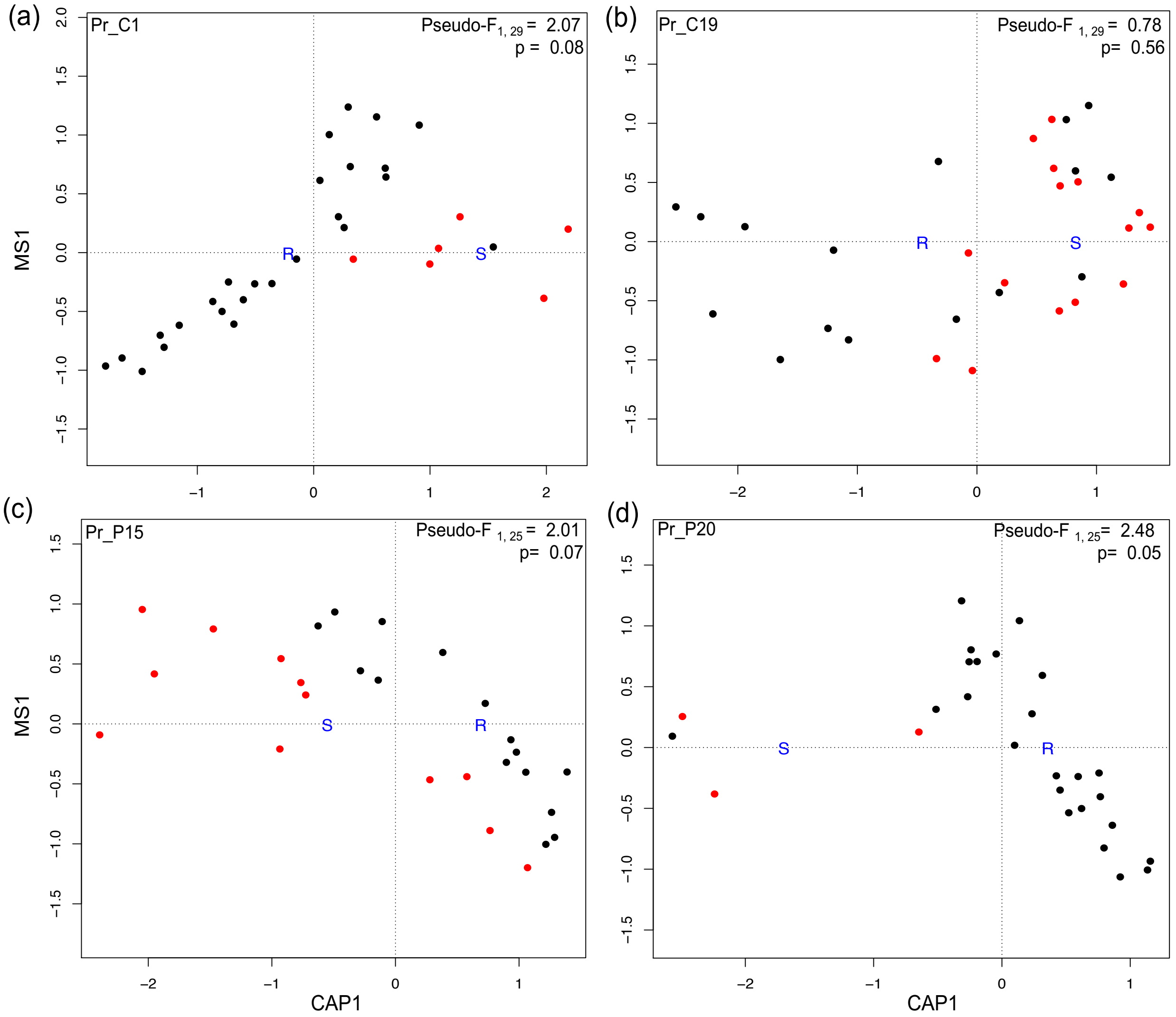

Supplement: Supplementary file 1 — Supplementary Materials [file 41598_2018_27681_MOESM1_ESM.pdf]
